# Mortality in Covid-19 patients hospitalized in a teaching hospital in Italy during the first 3 waves: Anna Odone

**DOI:** 10.1093/eurpub/ckac131.058

**Published:** 2022-10-25

**Authors:** M Bertolotti, M Betti, D Ferrante, F Giacchero, A Odone, G Franceschetti, M Carotenuto, G Pacileo, A Maconi

**Affiliations:** 1 Research Training Innovation Infrastructure, Alessandria, Italy; 2 Department of Translational Medicine, University of Eastern Piedmont, Novara, Italy; 3 Department of Public Health, University of Pavia, Pavia, Italy; 4 Medical Directorate, Alessandria, Italy; 5 Department of Integrated Activities Research, Local Health Company, Alessandria, Italy

## Abstract

**Introduction:**

In Italy a Covid-19 pandemic pattern was observed, characterized by several waves, with an excess total mortality of 178000 deaths. Alessandria, Italy is the Piedmont province with the highest proportion of mortality from Covid-19 in the first 4 months of 2020, compared to the rest of the region.

**Objectives:**

To analyze mortality in patients hospitalized for Covid-19 in the Alessandria Hospital (AO AL), considering the first 3 waves.

**Materials and methods:**

Subjects aged ≥18 with a diagnosis of Covid-19 admitted to the AO AL in the first 50 days of the first 3 waves were included. The first wave started on 24 February 2020 (first day of available data by the Ministry of Health), the second wave on 14 September 2020 (first day of the 2020/21 school year), the third wave on 15 February 2021 (peak of cases detected by the Italian College of Health). The causes of death were obtained from the National Institute of Statistics death cards and codified according to the International Classification of Diseases, 9th revision, classification.

**Results:**

We included 825 subjects (median age: 73 years; male prevalence: 60.7%). The subjects hospitalized in the first wave were 464, in the second wave 255, in the third wave 106. A total of 309 subjects died (37.5%), of which 218 in the first wave (70.6%), 69 in the second wave (22.3%), 22 in the third wave (7.1%). The most frequent causes of death were “Covid-19 pneumonia” (61.5%) and “respiratory distress syndrome” (19.4%). Death occurred after hospital discharge in 40% of cases. 6 months after admission, the survival rate was 53% among patients of the first wave, 73% and 78% for those of the second and third wave. Patients hospitalized in the first and second waves showed a greater risk of death compared to patients of the third wave (HR = 2.8; 95% CI 1.8-4.4 and HR = 1.4; 95% CI 0.8-2.2).

**Conclusions:**

Data showed a difference in mortality between the 3 waves with a statistically significant variation between the first and third waves.

**Key messages:**

• Data showed a difference in mortality between the 3 waves.

• Data showed a statistically significant variation in mortality between the first and third waves.

